# Evidence consistent with the multiple-bearings hypothesis from human virtual landmark-based navigation

**DOI:** 10.3389/fpsyg.2015.00488

**Published:** 2015-04-28

**Authors:** Martha R. Forloines, Kent D. Bodily, Bradley R. Sturz

**Affiliations:** ^1^Department of Psychology, Auburn University, Auburn, AL, USA; ^2^Department of Psychology, Georgia Southern University, Statesboro, GA, USA

**Keywords:** spatial learning, multiple bearings hypothesis, landmark learning, virtual navigation

## Abstract

One approach to explaining the conditions under which additional landmarks will be learned or ignored relates to the nature of the information provided by the landmarks (i.e., distance versus bearings). In the current experiment, we tested the ability of such an approach to explain the search behavior of human participants in a virtual landmark-based navigation task by manipulating whether landmarks provided stable distance, stable direction, or both stable distance and stable direction information. First, we incrementally shaped human participants’ search behavior in the presence of two ambiguous landmarks. Next, participants experienced one additional landmark that disambiguated the location of the goal. Finally, we presented three additional landmarks. In a control condition, the additional landmarks maintained stable distances and bearings to the goal across trials. In a stable bearings condition, the additional landmarks varied in their distances but maintained fixed bearings to the goal across trials. In a stable distance condition, the additional landmarks varied in their bearings but maintained fixed distances to the goal across trials. Landmark stability, in particular, the stability of landmark-to-goal bearings, affected learning of the added landmarks. We interpret the results in the context of the theories of spatial learning that incorporate the nature of the information provided by landmarks.

## Introduction

At a basic level, spatial learning involves the use of internal (e.g., dead reckoning) and external cues (e.g., landmarks) to navigate from one location (e.g., home) to another (e.g., food). At a more complex level, spatial learning involves the extraction of the relationships between locations (for a review, see Healy, 1998; Brown, 2006). Regardless of level of complexity, the study of landmark use for navigation has received considerable attention. Defined as learning about spatial relationships among objects in the environment, landmark learning may involve landmark-to-goal or landmark-to-landmark spatial relationships (Cheng, 1988, 1989, 1990, 1994; Sturz and Katz, 2009; Sturz et al., 2011; for a review, see Cheng and Spetch, 1998). Such learning has been studied in a variety of animals including bees, birds, rats, monkeys, and humans (for a review, see Brown, 2006; Kelly and Gibson, 2007), and extant research suggests that many animals are able to utilize landmarks for navigation between locations in the environment.

Numerous researchers have investigated the conditions under which landmarks will be learned or ignored. For example, Timberlake et al. (2007) provided evidence for disruption in landmark learning using a Morris Water Maze task with rats. Results showed that presentation of a pre-trained landmark that was fixed, proximal, and consistently predictive of the platform resulted in a decrement in search performance when rats were subsequently tested in the presence of an added distal landmark. Importantly, however, if the pre-trained landmark varied or was distal to the platform, an added proximal landmark resulted in an improvement in performance. Overall, the findings revealed that the proximity and stability of the landmark to the goal location affected subsequent learning of additional landmarks.

Similar results have been found by Biegler and Morris (1996) with rats in a square enclosure trained with fixed or variable positioned landmarks in relation to the distance from the goal. Rats that experienced fixed landmarks during training showed highly concentrated search around the goal, but rats that experienced varied landmarks during training showed substantial variability in search around the goal. Overall, these findings suggest that a lack of stability in landmark-goal relationships reduces learning about the landmarks and produces a decrement in search performance.

Sutton (2002) trained pigeons in the presence of distinct landmark configurations composed of four landmarks. After establishing that pigeons were utilizing the landmark configuration to determine the goal location, additional tests were conducted that displaced a single landmark from the configuration. However, these displacement tests did not disrupt search accuracy. Such results were interpreted as indicating that pigeons acquired information about distance and direction from all individual landmarks.

Similarly, a series of experiments by Sturz and Katz (2009) revealed that pigeons encoded spatial information from multiple environmental cues. Specifically, Sturz and Katz (2009) trained pigeons to locate the middle of a two-landmark array composed of two identical landmarks. Following training, control, contraction, and expansion tests revealed that pigeons continued to search in the middle of the array. Follow-up experiments revealed that training in the presence of a two-landmark array composed of two distinct landmarks did not disrupt learning about the individual landmarks composing the array. Specifically, pigeons were able to utilize the individual landmarks to determine relevant distance or directional information. Such results were also interpreted as indicating that pigeons acquired information about distance and direction from all individual landmarks.

Collectively, the aforementioned results can be interpreted in the context of theoretical approaches that relate to the extraction of distance and directional information from individual landmarks (see Kamil and Jones, 1997, 2000). Importantly, the use of both distance and directional information from multiple landmarks has also been suggested to be involved in the search behavior of nutcrackers (Kamil and Jones, 1997, 2000). Specifically, nutcrackers appear to be capable of learning complex geometric rules from landmarks such as middle, constant bearing, and constant distance (Kamil and Jones, 2000; see also, Jones et al., 2002; Spetch et al., 2003).

In an effort to account for the use of multiple landmarks during navigation, Kamil and Cheng (2001) proposed the *multiple bearings hypothesis* (MBH) which suggests that animals encode landmark-to-goal bearings from multiple landmarks. Such encoding of multiple bearings provides an opportunity for search behavior to be concentrated at the intersection of the individual bearings. Importantly, this area of potential search will decrease as the number of landmarks providing unique bearings to the goal increase. In short, any landmark that adds a unique bearing to the goal location is suggested to restrict the potential search area. From this perspective, landmarks that provide a unique bearing to the goal location will be learned but landmarks that do not provide a unique bearing to the goal will be ignored.

Kamil et al. (2001) tested and confirmed predictions of the MBH with Clark’s nutcrackers. Specifically, they tested the relationship between number of landmarks, interlandmark distance, and errors in searching for the goal. Kamil et al. (2001) found that a greater number of landmarks led to improved search accuracy. In addition, distance information was more error prone than direction information. Of note, distance errors increased with increasing interlandmark distances, but direction errors remained constant.

Most relevant to present purposes, both human children and adults are able to use individual landmarks or a landmark array to locate a goal location (Spetch, 1995; Spetch et al., 1996, 1997; Waller et al., 2000; Hartley et al., 2003; MacDonald et al., 2004; Foo et al., 2005; Sturz and Bodily, 2010; Sturz et al., 2011; Bodily et al., 2012). Following initial learning in the presence of landmarks or a landmark array, landmark manipulations reveal that humans appear to encode distance and direction information from multiple landmarks in much the same ways as described above for various birds.

For example, Bullens et al. (2010) tested human adults and children on their ability to use proximal and distal cues in an object replacement procedure. Importantly, the position of the goal remained consistent relative to distal cues, but the position of proximal cues varied in across trials. Overall, both groups successfully located the goal, and there were no differences from chance in task completion across age groups; however, children performed worse than adults. Performance differences emerged with respect to how the age groups located the goal. Adults tended to rely on the more stable distal landmarks while children tended to rely on the proximal cues. Further, errors in distance and angle to the goal were calculated and revealed that adults showed more precise angular estimates of the goal while children were more precise in distance estimates to the goal. These results agree with previous animal studies of landmark stability and proximity to the goal. Specifically, human adults perform similarly to the rats in the aforementioned study by Timberlake et al. (2007) in that they relied on the most stable and informative landmarks in relation to the goal.

Given its potential to explain the conditions under which landmarks will be learned versus ignored (Kamil and Cheng, 2001), the MBH may be a viable candidate to explain the search behavior of human participants in a landmark-based navigation task. To that end, the present experiment employed a three-phase spatial learning paradigm that varied the stability of landmarks across groups. In an initial phase, we incrementally shaped human participants’ goal-locating behavior in the presence of a landmark array composed of two identical and stable landmarks; however, the location of the goal was directionally ambiguous with respect to east and west of the landmark array. This phase was intended to introduce participants to the task. Next, participants experienced one additional stable landmark that disambiguated the location of the goal. Finally, we presented three additional landmarks. Specifically, participants were randomly assigned to one of three groups that that differed with respect to the stability of the landmark presentations. For participants in the Control group, landmarks maintained fixed distances and bearings to the goal across trials. For participants in the Stable Bearings group, the landmarks varied in their distances but maintained fixed bearings to the goal across trials. For participants in the Stable Distance group, the landmarks varied in their bearings but maintained fixed distances to the goal across trials. It is important to note that the Stable Bearings and Stable Distance groups experienced an identical number of stable landmark-goal relationships. The only difference between the groups was the type of stable relationship. Interspersed within these trials, we probed the extent of learning about the added landmarks during critical test trials in which we either presented only the added array or the initial training array in combination with each of the added landmarks. The purpose of the Added Array trials was to assess the extent to which learning occurred about landmarks introduced after initial learning. The purpose of the Individual Landmark trials was to assess the extent to which learning would occur about individual landmarks composing the Added Landmark array. As importantly, such tests allowed us to determine the extent to which learning about the Added Array and Individual Landmarks differed across groups.

According to the MBH (Kamil and Cheng, 2001), if novel landmarks provide additional bearing information regarding the location of the goal, these landmarks should be learned. Given that the Added Array and Individual Landmarks provided additional bearing information to the goal for the Control and Stable Bearings groups but not the Stable Distance group, information about these landmarks should be learned by the Control and Stable Bearings groups but not by the Stable Distance group. Specifically, performance on the Added Array trials and Individual Landmark trials should not differ from that of the baseline trials for the Control and Stable Bearing groups, but performance during these trial types should be inferior to baseline for the Stable Distance group. Moreover, the Control and Stable Bearing groups should perform superior to the Stable Distance group because only under those conditions do the novel landmarks provide additional and reliable bearing information regarding the location of the goal.

## Materials and Methods

### Participants

Thirty-six undergraduates (20 male and 16 female) participated in this study. The participants were recruited from Psychology courses and awarded with either class credit or extra credit for their participation.

### Apparatus

An interactive, three-dimensional (3D) virtual environment was created using Valve Hammer Editor and run on the Half-Life Team Fortress Classic platform. A personal computer with a triple display flat screen monitor (2400 × 600 pixels, with a field of view of 115°) and speakers served as the interface for the virtual environment. Participants experienced the virtual environment in first-person perspective. A Logitech Dual Action gamepad was used to navigate and make a selection in the virtual environment. The left joystick allowed for navigation (forward, backward, left, and right). Any button on the right side of the gamepad (1–4) could be pressed to make a selection. Data were collected and recorded with Half-Life Dedicated Server on a similar computer.

### Stimuli

**FIGURE 1:**
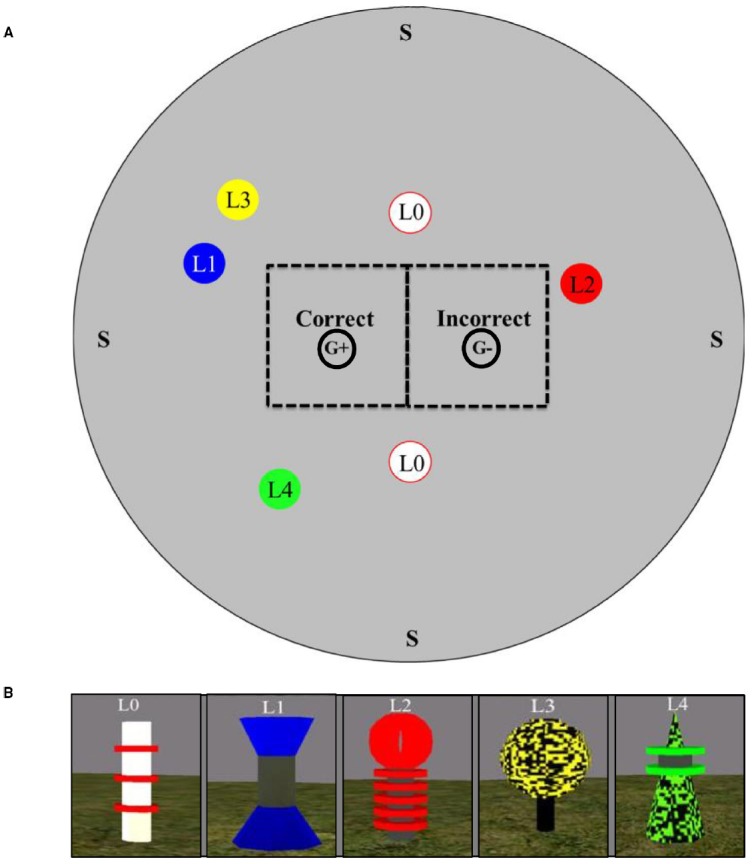
**(A)** Overhead view of the virtual environment search space illustrating the location of all landmarks. Please note that the presence of all landmarks is for illustrative purposes only—landmarks were never presented simultaneously to participants (see text for details). Please also note that the location of Landmark 3 (L3) and Landmark 4 (L4) illustrate only one potential location depending on group (see text for details). For illustrative purposes, potential start locations are indicated with an “S”. Also for illustrative purposes, the areas designated as Correct and Incorrect locations for data analytic purposes are indicated with dashed squares and marked with “Correct” and “Incorrect.” Finally, for illustrative purposes, the goal location (G+) and its mirrored location (G–) (see text for details). Please note figure is not to scale. **(B)** Screen shots of the individual landmarks used in the virtual environment search task. L0, Landmark 0; L1, Landmark 1; L2, Landmark 2; L3, Landmark 3; L4, Landmark 4.

**FIGURE 2:**
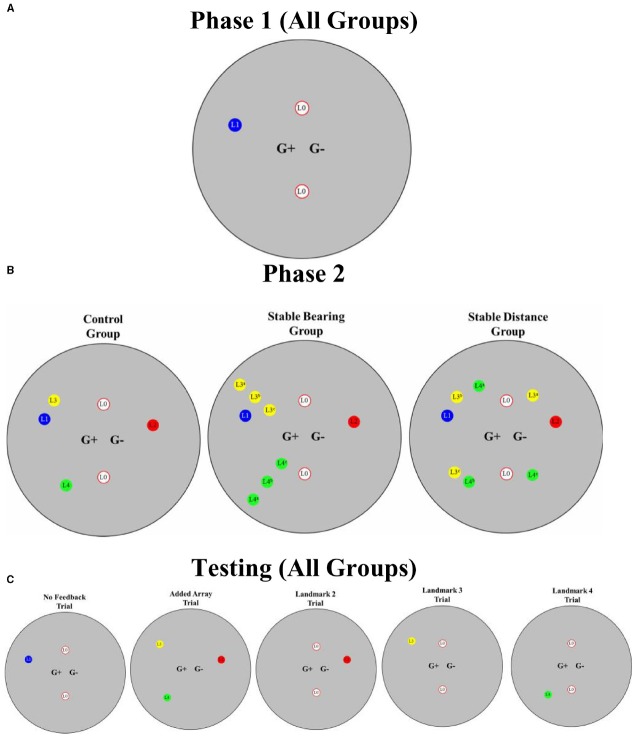
**(A)** Overhead view illustrating initial training (Phase 1) for all groups. **(B)** Phase 2 training for each group. **(C)** Testing for all groups. Please note that each of the potential locations of Landmark 3 (L3^a^, L3^b^, L3^c^) and Landmark 4 (L4^a^, L4^b^, L4^c^) is illustrated **(B)** even though each landmark occupied only one location that varied according to constant distance (Stable Distance Group) or constant bearing (Stable Bearing Group). Please note that figure is not to scale.

The experimental room [1424 virtual units (vu) × 1424 vu with 712 vu radius; 1 vu = ∼2.54 cm] was round and consisted of solid gray walls, green floor, and a black ceiling. A circular area with a radius of 64 vu was designated as the goal location (marked “G+” in Figure 1A). Five landmarks served as stimuli. All landmarks were identical in height at 76 vu and varied in width from 15 vu to 36 vu (see Figure 1B). Two landmarks [Landmark 0 (L0)] were white with three red stripes; all stripes were located horizontally around the cylinder in the midsection. Landmark 1 (L1) was an hourglass shape with a blue base and top with a dark gray cylinder in the midsection; each portion of the landmark was the same height. Landmark 2 (L2) was a black thin cylinder with a red sphere located on top. Around the bottom half of L2 were five red circular terraces surrounding the black base. Landmark 3 (L3) was a black thin cylindrical base with a yellow and black pixilated sphere on the top. Landmark 4 (L4) was a green and black pixilated pyramid with a pair of square terraces on the top portion of the pyramid (see Figures 1 and 2 for landmark layouts). In the middle of the terraces was a black filling. For some trials (see below), a semi-transparent green sphere (64 vu) was present.

### Procedure

Participants were randomly assigned to one of three groups (Control, Stable Distance, Stable Bearing). The experiment consisted of 43 total trials (36 training trials and seven test trials interspersed). Training consisted of three phases Phase 0, Phase 1, and Phase 2. Participants began each trial randomly placed at one of four start locations at the edge of the room facing toward the wall (all marked “S” in Figure 1A). A low tone played to signify the trial had begun. Participants were instructed to find the goal location which was marked by the semi-transparent green sphere (marked “G+” in Figure 1A). Feedback concerning accurate goal localization differs across phases (see below).

### Training

#### Phase 0

Trials 1 through 5 were intended to introduce participants to the task and consisted of a two-landmark array (L0, see Figure 2A). Although the area designated as the goal location was present and stable on all these trials, the two stable landmarks were an ambiguous indicator of the east or west location of the goal because the array was located in a feature-less cylindrical room. As a result, participants were not able to discern whether the goal location was located on the east or west side of the landmark array. One landmark was located 22.5° from north and the other was located 157.5° from north. Both landmarks were located 236 vu from the goal location.

We incrementally shaped participants’ search for the goal location over the course of the first five trials. A semi-transparent green sphere marking the goal location became visible when participants crossed an invisible circle (1024 vu in diameter) centered on the goal location during the first trial. The diameter of this area successively decreased in size on trials 2–4 from 428 vu, to 248 vu, to 128 vu. Trial 5 also had an area of 128 vu to activate the green sphere and required the participants to remain in the goal location for a duration of 1 s. For all trials, participants were required to navigate to the marker with the left joystick and press one of the right gamepad buttons to indicate they had located the goal. Pressing one of the right gamepad buttons terminated the trial, and started a 5 s inter-trial interval (ITI) during which the screen was black.

#### Phase 1

Trials 6 through 15 consisted of the two landmarks from Phase 0 (L0s) with the addition of a disambiguating landmark (L1). L1 was positioned west of the line bisecting the L0 array 292.5° from north and 354 vu from the goal location. This three-landmark array allowed for precise localization of the goal location by providing directional information from the L0 array (see Figure 2A).

During Phase 1, if participants remained in the goal location for a duration of 3 s, the green marker appeared and an auditory “ding” sound occurred. Both the visual and auditory feedback were provided to inform participants they were situated in the goal location. If a participant did not remain in the goal location for the required 3 s duration before 45 s had elapsed, then the semi-transparent green sphere appeared at the goal location. In both cases, once the semi-transparent green sphere appeared, participants were required to navigate to the green sphere and press any of the gamepad buttons to terminate the trial and initiate the 5 s ITI. A low tone signified the start of each trial. Two no feedback trials occurred during Phase 1 at Trial 11 and 15 to provide participants with goal absent experiences, assess learning of the initial landmark array, and serve as baseline comparisons for test trials (see below). For the No Feedback trials, we removed the goal location and terminated the trial after a duration of 30 s regardless of how long participants remained at the goal location.

#### Phase 2

In addition to the presence of the three-landmark array from Phase 1 (L0, L0, L1), trials 16 through 43 also consisted of the presence of three additional landmarks (L2, L3, and L4). As a result, there were six landmarks present on these trials. L2 was located 67.5° from north and 354 vu from the goal location. L3 was located 315° from north and 354 vu from the goal location. L4 was located 202.5° from north and 354 vu from the goal location. For the Control group, all landmarks were stable in their location for the duration of Phase 2. For the Stable Bearing group, the distance of L3 and L4 from the goal location randomly varied between 472, 354, and 236 vu from trial-to-trial but the angles formed to the goal location remained constant from trial-to-trial. For the Stable Distance group, the angles formed by L3 and L4 to the goal location randomly varied between 337.5°, 315°, and 135° (L3) and between 225°, 202.5°, and 45° (L4) from trial-to-trial but the distances to the goal location remained constant from trial-to-trial (see Figure 2B).

### Testing

As mentioned, two no feedback trials were interspersed into Phase 1 (Trials 11 and 15). In addition to these two No Feedback trials, we interspersed seven test trials throughout Phase 2. For all of the seven test trials, we removed the goal location and terminated the trial after a duration of 30 s regardless of how long participants remained at the goal location. Testing consisted of two basic trial Types: (1) Added Array trials and (2) Individual Landmark trials. The purpose of the Added Array trials was to assess the extent to which learning would occur about landmarks introduced after initial learning. The purpose of the Individual Landmark trials was to assess the extent to which learning would occur about individual landmarks composing the added landmark array. For the Added Array trials, the initial training two-landmark array (L0s) was removed, and the three landmarks introduced during Phase 2 (L2, L3, L4) were presented simultaneously. For Individual Landmarks trials, each of the three landmarks introduced during Phase 2 (L2, L3, L4) were presented individually in conjunction with the initial two-landmark array (L0s). For all test trials, the landmarks were positioned in a manner identical to the location of the landmarks for the Control group during Phase 2 training (see Figure 2B) to allow comparisons across groups. Test trials were interspersed pseudo-randomly throughout Phase 2 such that one test trial occurred within each four-trial block; as a result, each participant experienced a total of seven test trials. Test trials were presented in the same order for all participants. Please refer to Figure 2C for a summary of all test trials.

## Results

Participant locations were recorded in coordinates (x, y) once per second throughout each 30-s test trial. Given that the task was a landmark-based navigation task and that participants were trained to respond near landmarks, we assumed participants would search near landmarks regardless of trial type. As a result, we partitioned the search space into two identical squares (320 vu × 320 vu) located on either side of the line bisecting the two L0 landmarks to determine whether participants were able to accurately locate the goal location (refer to Figure 1A). The area of the square on the west side overlapped where the goal location would have been located and was designated as Correct (area = 102,400 vu × 102,400 vu). The area of the square on the east side overlapped where the mirrored location of the goal and was designated as Incorrect (area = 102,400 vu × 102,400 vu). Refer to Figure 1A for an illustration of Correct (G+) and Incorrect areas (G–). To illuminate search performance, we measured the relative amount time spent in Correct and Incorrect areas (preference ratio). The preference ratio was defined as

$$Preference=\frac{T_{C}}{T_{C}+T_{I}}$$

where *T_C_* is the time in the Correct area and *T_I_* is the time in the Incorrect area. Importantly, utilizing this preference ratio also allowed for an *a priori* chance value of 0.5. In short, a preference ratio value of 0.5 would indicate an inability to discern the difference between the east and west sides of the L0 array.

### Training

#### Phase 0

**FIGURE 3:**
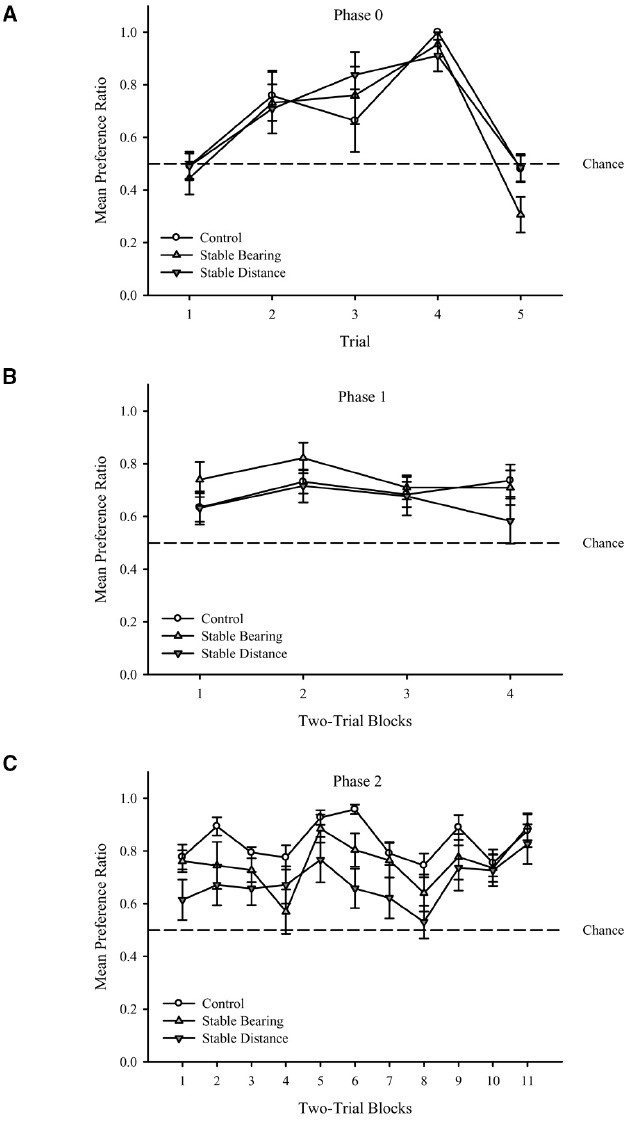
**(A)** Mean preference ratio during Phase 0 **(A)**, Phase 1 **(B)**, and Phase 2 **(C)** training trials for each group. Dashed lines represent chance performance (0.5). Error bars represent standard errors of the means.

As shown in Figure 3A, the shaping procedure resulted in an increase in preference ratios across initial trials with the exception of Trial 5. Importantly, performance did not differ across groups, and performance was greater than chance levels for most trials. These results were confirmed with a two-way mixed analysis of variance (ANOVA) conducted on preference ratio^1^ with Group (Control, Stable Bearing, Stable Distance) and Trial (1–5) as factors and revealed a main effect of Trial, *F*(4,116) = 22.62, *p* < 0.001. Neither the effect of Group, *F*(2,29) = 0.63, *p* = 0.54, nor the interaction, *F*(8,348) = 0.72, *p* = 0.67, was significant. One-sample *t*-tests comparing mean preference ratio (see text footnote 1) for each trial (collapsed across group) to chance performance (0.5) revealed that the mean preference ratio for Trial 1 was not significantly different from chance, *t*(31) = 0.79, *p* = 0.43, but Trial 2–4 were significantly above chance, *t*s(31) > 4.05, *p*s < 0.001. Trial 5 was significantly below chance, *t*(31) = 2.2, *p* < 0.05. We suspect that the drop in performance during Trial 5 was a result of this trial being the first time participants were required to remain in the goal location for a duration of 1 s to activate the green sphere.

#### Phase 1

As shown in Figure 3B, performance was stable across training trials for all groups. As importantly, performance did not differ across groups, and performance was greater than chance levels for all trial blocks. These results were confirmed with a two-way mixed ANOVA conducted on preference ratio with Group (Control, Stable Bearing, Stable Distance) and Trial Block (1–4) as factors and revealed no main effect of Group, *F*(2,33) = 1.93, *p* = 0.16, no main effect of Trial Block, *F*(3,99) = 1.35, *p* = 0.26, and no significant interaction, *F*(6,99) = 0.50, *p* = 0.80. One-sample *t*-tests comparing mean preference ratio for each Trial Block (collapsed across group) to chance performance (0.5) revealed that the mean preference ratio for all trial blocks was significantly above chance *t*s(35) > 4.22, *p*s < 0.001.

#### Phase 2

As shown in Figure 3C, performance fluctuated across training trials for all groups. Importantly, however, performance did not differ across groups, and performance was greater than chance levels for all trial blocks. These results were confirmed with a two-way mixed ANOVA conducted on preference ratio with Group (Control, Stable Bearing, Stable Distance) and Trial Block (1–11) as factors and revealed a main effect of Trial Block, *F*(10,330) = 7.11, *p* < 0.001. Neither the effect of Group, *F*(2,33) = 3.18, *p* = 0.06, nor the interaction, *F*(20,330) = 1.24, *p* = 0.22, was significant. One-sample *t*-tests comparing mean preference ratio for each Trial Block (collapsed across group) to chance performance (0.5) revealed that the mean preference ratio for all trials blocks was significantly above chance *t*s(35) > 3.77, *p*s < 0.01.

#### No Feedback

**FIGURE 4:**
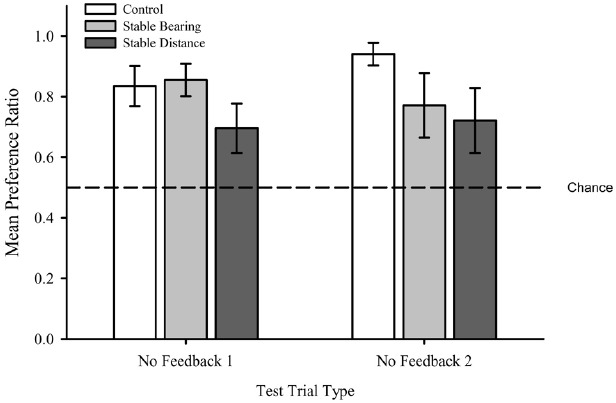
**Mean preference ratio during No Feedback trials for each group.** Dashed line represents chance performance (0.5). Error bars represent standard errors of the means.

As shown in Figure 4, the Control (*M* = 0.89, SEM = 0.04), Stable Bearing (*M* = 0.81, SEM = 0.06), and Stable Distance (*M* = 0.71, SEM = 0.08) groups did not differ with respect to their relative allocation of time in the areas designated as Correct versus Incorrect on No Feedback Trials. In addition, this measure of performance did not differ across No Feedback Trial 1 (*M* = 0.80, SEM = 0.04) and No Feedback Trial 2 (*M* = 0.81, SEM = 0.05). Importantly, these values were greater than what would be expected on the basis of chance. These results were confirmed with a two-way mixed ANOVA on mean preference ratio^2^ with Group (Control, Stable Bearing, Stable Distance) and No Feedback Trial (1, 2) as factors which revealed no main effect of Group, *F*(2,32) = 1.92, *p* = 0.15, no main effect of Trial Type *F*(1,32) = 0.09, *p* = 0.77, and no interaction *F*(2,32) = 1.04, *p* = 0.37. One-sample *t*-tests, confirmed that the mean preference ratios (see text footnote 2; collapsed across group) were greater than chance (0.5) for No Feedback Trial 1, *t*(34) = 7.55, *p* < 0.001, and No Feedback Trial 2, *t*(34) = 6.00, *p* < 0.001.

Collectively, these analyses suggest that all participants were capable of determining the location of the goal to an equivalent level at above chance levels during shaping and training and that performance remained stable across No Feedback trials. Given that both measures of search performance did not statistically differ by the second presentation of the No Feedback trials, we collapsed performance across No Feedback trials (Mean No Feedback) for all remaining analyses. Such a measure served as an indicator of baseline performance for comparisons to Added Array and Individual Landmark tests.

## Testing

**FIGURE 5:**
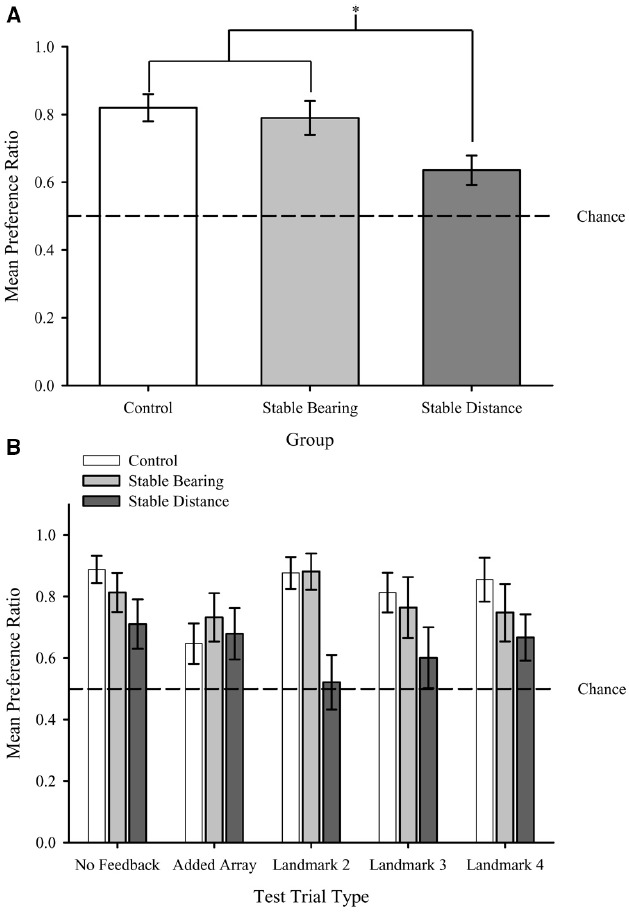
**(A)** Mean preference ratio plotted by Group collapsed across Test Trial Types (main effect of Group). **(B)** Mean preference ratio during Testing plotted by Trial Type for each Group. Dashed lines represent chance performance (0.5). Error bars represent standard errors of the means. *p < 0.05.

In order to determine the extent of learning about the added landmarks during critical test trials in which we either presented only the added array or the initial training array in combination with each of the added landmarks, we compared these trials types to the No Feedback (i.e., baseline) trials both within and across groups. Such comparisons allowed us to determine similarities and differences in learning about the added array and individual landmarks for each group. An omnibus Group (Control, Stable Bearing, Stable Distance) × Test Trial Type (Mean No Feedback, Added Array, L2, L3, L4) mixed measures ANOVA was conducted on preference ratio^3^ and revealed only a main effect of Group, *F*(2,32) = 4.47, *p* < 0.05. Neither the effect of Test Trial Type, *F*(4,128) = 1.24, *p* = 0.29, nor the interaction, *F*(8,128) = 1.41, *p* = 0.19, was significant. Figure 5A shows the main effect of Group with mean preference ratio plotted by Group. Fisher’s least significant difference (LSD) *post hoc* tests on the Group factor revealed that the mean preference ratios for the Control and Stable Bearing groups were significantly greater than that of the Stable Distance (*p*s < 0.05). The mean preference ratios for the Control and Stable Bearings groups did not differ (*p* = 0.66).

As shown in Figure 5B, preference ratios (see text footnote 3) for all groups were significantly above that which would be expected by chance (0.5) during No Feedback, *t*s(11/10) > 2.6, *p*s < 0.05 Importantly, however, group differences emerged with respect to what would be expected on the basis of chance during the Added Array and Individual Landmark Tests. Specifically, the mean preference ratio for both the Control and Stable Bearings groups were significantly above chance for the Added Array trials and all Individual Landmark tests, *t*s(11) > 2.2, *p*s < 0.05, but none of the preference ratios for the Stable Distance group were significantly above chance for the Added Array or any of the Individual Landmark tests, *t*s(10) < 2.2, *p*s > 0.05. Collectively, results suggest that information from the Added Array and Individual Landmarks was learned by both the Control and Stable Bearings groups but not by the Stable Distance group. Please see Table 1 for a summary of the comparisons of mean preference ratios to chance levels by Group and Test Trial Type.

**TABLE 1:** **Comparisons of preference ratio (time in area designated as correct/total time in area designated as correct and incorrect) relative to chance (0.5) for each group by test trial type**.

	Test trial type				
	No feedback	Added array	Landmark 2	Landmark 3	Landmark 4
**Group**					
Control	Above	Above	Above	Above	Above
Stable bearing	Above	Above	Above	Above	Above
Stable distance	Above	Equal	Equal	Equal	Equal

Above p < 0.05, Equal p > 0.05. The mean preference ratio collapsed across the above trial types indicated that the mean preference ratio for the control and stable bearing groups were significantly greater than that of the stable distance group. The mean preference ratio for control and stable bearings groups were not significantly different from each other.

## Discussion

Results from initial shaping and training revealed that participants in all three groups were able to learn to determine the goal location. Despite fluctuations in performance during Phase 2, performance was above chance levels and did not differ across groups for any of the phases of training. As importantly, performance during the No Feedback trials indicated that participants in all groups learned the location of the goal to a similar extent, and performance during the No Feedback trials was significantly above chance levels. Such results indicate that participants in all three groups were able to utilize the landmarks from initial training to determine the goal location. Although no differences emerged in performance across Test Trial Types during Testing, Group differences did emerge such that the Control and Stable Bearing groups performed superior to that of the Stable Distance group. Group differences also emerged with respect to the Added Array and Individual Landmark tests such that participants in the Control and Stable Bearings groups performed above chance levels on all of these trial types whereas participants in the Stable Distance group performed at chance levels on all of these trial types. In short, performance during the Added Array and Individual Landmark tests revealed that both the Control and Stable Bearing groups learned about the array and the individual landmarks composing the array, but participants in the Stable Distance group were unable to utilize either the landmark array or any of the individual landmarks composing the array to determine the goal location.

Of empirical importance, our results appear consistent with prior research indicating the importance of landmark stability (Biegler and Morris, 1996; Timberlake et al., 2007). Of theoretical importance, our results appear to be consistent with a multiple bearings account of landmark use (Kamil and Cheng, 2001). Specifically, our results reveal conditions under which human participants will learn or ignore additional landmarks. More specifically, if landmark movement involves variations in distance (while holding bearings constant), these landmarks appear to be learned. In contrast, if landmark movement involves variations in bearings (while holding distance constant), these landmarks appear to be ignored. In short, the similarity in performance for the Control and Stable Bearings group coupled with the superior performance of both of these groups compared to the Stable Distance group indicates that landmark stability is important but only with respect to landmark-to-goal bearings in human landmark-based navigation. Thus, consistent with the MBH and prior research with non-human species (Sutton, 2002; Sturz and Katz, 2009), humans also appear to learn landmarks provided they indicate a unique bearing to the goal.

We acknowledge that one limitation of the present study relates to the continued presence of the two-landmark array during individual landmark tests. In retrospect, individual landmarks tests in the absence of the two-landmark array may have been more theoretically diagnostic as they would have allowed the comparison of distance measures from the goal location within and across groups. Such distance measures would have also allowed us to separate distance error from direction error to more specifically isolate the contribution of these sources of spatial information to goal localization. As the two-landmark array was present during the individual landmark tests, the individual landmarks could only serve to disambiguate the correct from the incorrect side of the array itself. As such, the present task resembled more of a discrete choice between two locations as opposed to a continuous measure of spatial behavior.

We also acknowledge the limitation that the initial learning condition in the presence of the two-landmark array may have provided more reliable distance information compared to the ambiguous directional information. As a result, directional information was required to disambiguate the correct from the incorrect side of the array, and this requirement may have inadvertently created stronger reliance on the use of bearings in the present task. Such an interpretation is somewhat supported by visual inspection of training data in that performance of the Stable Distance group was inferior to that of Control and Stable Bearing groups. As a result, such reliance on bearings may be responsible for the inferior performance of the Stable Distance group during Testing (or superior performance by the Stable Bearings group) than might otherwise have been observed. One potential way of addressing such a limitation would be to compare the original training in the presence of the two landmark array with a condition involving ambiguous distance information. Such a comparison may increase the likelihood of participants relying on distance as opposed to directional information.

Despite these limitations, results from the Added Array and Individual landmark tests indicated that participants in the Control and Stable Bearings groups were able to discern the correct from the incorrect side of the two-landmark array whereas participants in the Stable Distance group were unable to discern the correct from the incorrect side of the two-landmark array. As a result, it seems especially clear that participants were able to utilize the array and individual landmarks composing the array when the provided stable bearing information to the goal location. In short, our results appear consistent with a multiple-bearings hypothesis in that novel landmarks will be learned provided they add additional bearing information regarding the location of the goal (Kamil and Cheng, 2001). Within a comparative context, this suggests that human landmark-based navigation may be capable of being accounted for by the same mechanisms used to explain landmark-based navigation of other species. Future research should continue to explore the ability of MBH to account for landmark-based navigation in other species to illuminate similarities and differences across mobile animals regarding the mechanisms underlying landmark-based navigation.

### Conflict of Interest Statement

The authors declare that the research was conducted in the absence of any commercial or financial relationships that could be construed as a potential conflict of interest.

## Footnotes

1 One participant in the Control Group, one participant in the Stable Bearing group, and two participants in the Stable Distance group were removed from analyses due to a lack of movement during a trial.

2 One participant in the Stable Distance group was removed from No Feedback Trial 1 analyses due to a lack of movement during the trial.

3 One participant in the Stable Distance group was removed from these analyses due to a lack of movement during the L2 test.

## References

[B1] Biegler R. Morris R. G. M. (1996). Landmark stability: Further studies pointing to a role in spatial learning. Q. J. Exp. Psychol. 49, 307–345. 8962538 10.1080/713932636

[B2] Bodily K. D. Daniel T. A. Sturz B. R. (2012). The roles of beaconing and dead reckoning in human virtual navigation. Learn. Motiv. 43, 14–23 10.1016/j.lmot.2012.01.002

[B3] Brown M. F. (2006). “Abstracting spatial relations among goal locations,” in Animal Spatial Cognition: Comparative, Neural, and Computational Approaches, eds Brown M. F. Cook R. G. (On-line). Available at: www.pigeon.psy.tufts.edu/asc/brown/

[B4] Bullens J. Nardini M. Doeller C. F. Braddick O. Postma A. Burgess N. (2010). The role of landmarks and boundaries in the development of spatial memory. Dev. Sci. 13, 170–180. 10.1111/j.1467-7687.2009.00870.x 20121873

[B5] Cheng K. (1988). Some psychophysics of the pigeon’s use of landmarks. J. Comp. Physiol. A 162, 815–826. 10.1007/BF00610970 3397923

[B6] Cheng K. (1989). The vector sum model of pigeon landmark use. J. Exp. Psychol. Anim. Behav. Process. 15, 366–375 10.1037/0097-7403.15.4.366

[B7] Cheng K. (1990). More psychophysics of the pigeon’s use of landmarks. J. Comp. Physiol. A 166, 857–864 10.1007/BF00187333 3397923

[B8] Cheng K. (1994). The determination of direction in landmark-based spatial search in pigeons: a further test of the vector sum model. Anim. Learn. Behav. 22, 291–301 10.3758/BF03209837

[B9] Cheng K. Spetch M. L. (1998). “Mechanisms of landmark use in mammals and birds,” in Spatial Representation in Animals, ed. Healy S. (Oxford: Oxford University Press), 1–17.

[B10] Foo P. Warren W. H. Duchon A. Tarr M. J. (2005). Do humans integrate routes into a cognitive map? Map- versus landmark-based navigation of novel shortcuts. J. Exp. Psychol. Learn. Mem. Cogn. 31, 195–215. 10.1037/0278-7393.31.2.195 15755239

[B11] Hartley T. Maguire E. A. Spiers H. J. Burgess N. (2003). The well-worn route and the path less traveled: distinct neural bases of route following and wayfinding in humans. Neuron 37, 877–888. 10.1016/S0896-6273(03)00095-3 12628177

[B12] Healy S. (1998). Spatial Representation in Animals. Oxford: Oxford University Press.

[B13] Jones J. E. Antoniadis E. Shettleworth S. Kamil A. (2002). A comparative study of geometric rule learning by nutcrackers (*Nucifraga columbiana*), pigeons (*Columba livia*), and jackdaws (*Corvus monedula*). J. Comp. Psychol. 116, 350–356. 10.1037/0735-7036.116.4.350 12539930

[B14] Kamil A. C. Cheng K. (2001). Way-finding and landmarks: the multiple bearings hypothesis. J. Exp. Biol. 2043, 103–113. 11104714 10.1242/jeb.204.1.103

[B15] Kamil A. C. Goodyear A. J. Cheng K. (2001). The use of landmarks by Clark’s nutcrackers: first tests of a new model. J. Navigation 54, 429–435 10.1017/S0373463301001436

[B16] Kamil A. C. Jones J. E. (1997). The seed-storing corvid Clark’s nutcracker learns geometric relationships among landmarks. Nature 390, 276–279 10.1038/36840

[B17] Kamil A. C. Jones J. E. (2000). Geometric rule learning by Clark’s nutcrackers (*Nucifraga columbiana*). J. Exp. Psychol. Anim. Behav. Process. 26, 439–453. 10.1037/0097-7403.26.4.439 11056884

[B18] Kelly D. M. Gibson B. M. (2007). Spatial navigation: spatial learning in real and virtual environments. Comp. Cogn. Behav. Rev. 2, 111–124.

[B19] MacDonald S. E. Spetch M. L. Kelly D. M. Cheng K. (2004). Strategies in landmark use by children, adults, and marmoset monkeys. Learn. Motiv. 35, 322–347 10.1016/j.lmot.2004.03.002

[B20] Spetch M. L. (1995). Overshadowing in landmark learning: Touch-screen studies with pigeons and humans. J. Exp. Psychol. Anim. Behav. Process. 21, 166–181. 7738499 10.1037//0097-7403.21.2.166

[B21] Spetch M. L. Cheng K. MacDonald S. E. (1996). Learning the configurations of a landmark array: I. Touch-screen studies with pigeons and humans. J. Comp. Psychol. 110, 55–68. 10.1037/0735-7036.110.1.55 8851553

[B22] Spetch M. L. Cheng K. MacDonald S. E. Linkenhoker B. A. Kelly D. M. Dooerkson S. (1997). Learning the configurations of a landmark array in pigeons and humans: II. Generality across search tasks. J. Comp. Psychol. 111, 14–24 10.1037/0735-7036.111.1.14

[B23] Spetch M. C. Rust T. B. Kamil A. Jones J. E. (2003). Searching by rules: Pigeons’ (*Columba livia*) landmark-based search according to constant bearing or constant distance. J. Comp. Psychol. 117, 123–132. 10.1037/0735-7036.117.2.123 12856782

[B24] Sturz B. R. Bodily K. D. (2010). Encoding of variability of landmark-based spatial information. Psychol. Res. 74, 560–567. 10.1007/s00426-010-0277-4 20177902

[B25] Sturz B. R. Cooke S. P. Bodily K. D. (2011). Solving for two unknowns: an extension of vector-based models of landmark-based navigation. J. Exp. Psychol. Anim. Behav. Process. 37, 368–374. 10.1037/a0022938 21744982

[B26] Sturz B. R. Katz J. S. (2009). Learning of absolute and relative distance and direction from discrete visual landmarks by pigeons (*Columba livia*). J. Comp. Psychol. 123, 90–113. 10.1037/a0012905 19236148

[B27] Sutton J. E. (2002). Multiple-landmark piloting in pigeons (*Columba livia*): landmark configuration as a discriminative cue. J. Comp. Psychol. 116, 391–403. 10.1037/0735-7036.116.4.391 12539935

[B28] Timberlake W. Sinning S. Leffel J. K. (2007). Beacon training in a water maze can facilitate and compete with subsequent room cue learning in rats. J. Exp. Psychol. Anim. Behav. Process. 33, 225–243. 10.1037/0097-7403.33.3.225 17620023

[B29] Waller D. Loomis J. M. Golledge R. G. Beall A. C. (2000). Place learning in humans: the role of distance and direction information. Spat. Cogn. Comput. 2, 333–354 10.1023/A:1015514424931

